# Beta-Blockers in Heart Failure With Reduced Ejection Fraction: A Systematic Review of Randomized Controlled Trials

**DOI:** 10.7759/cureus.91266

**Published:** 2025-08-29

**Authors:** Sara Elmobark, Fouad Hamad, Lemia Gameil Hassan Mutwali, Nora Qassem Alsyed Ali Mohamed Zain, Zainab Mohamed, Tartel Abdelhamed Mohamed Ahmed, Othman Awad Hassan Almzaini

**Affiliations:** 1 Internal Medicine, Aberdeen Royal Infirmary, Aberdeen, GBR; 2 Internal Medicine, University Hospital Galway, Galway, IRL; 3 Primary Health Care, Oman Ministry of Health, Muscat, OMN; 4 General Medicine, SEHA Salma Rehabilitation Hospital, Abu Dhabi, ARE; 5 General Medicine, Heartlands Hospital, Birmingham, GBR; 6 Internal Medicine, Nizwa Hospital, Nizwa, OMN; 7 Cardiology, Royal Papworth Hospital, Bedfordshire, GBR

**Keywords:** beta-blockers, heart failure with reduced ejection fraction, myocardial infarction, preserved ejection fraction, randomized controlled trials, systematic review

## Abstract

Heart failure with reduced ejection fraction (HFrEF) remains a significant global health burden, with beta-blockers serving as a cornerstone therapy due to their ability to modulate neurohormonal activation and improve outcomes. However, their efficacy in post-myocardial infarction (MI) patients with preserved ejection fraction (EF) remains debated. This systematic review evaluates the clinical effects of beta-blockers in HFrEF and post-MI populations to clarify their role across different patient phenotypes. Eight randomized controlled trials (RCTs) were systematically reviewed following Preferred Reporting Items for Systematic Reviews and Meta-Analyses (PRISMA) guidelines. Eligible studies assessed beta-blockers in HFrEF or post-MI patients, reporting outcomes such as mortality, hospitalizations, or changes in EF. Data were extracted independently by two reviewers, and risk of bias was assessed using the Cochrane RoB 2 tool. A narrative synthesis was performed due to heterogeneity in study designs and interventions. Beta-blockers demonstrated significant reductions in all-cause mortality and heart failure hospitalizations in chronic HFrEF patients. In contrast, they showed neutral effects on mortality and cardiovascular outcomes in post-MI patients with preserved EF. Safety profiles were favorable, with no excess adverse events. Methodological quality was high, with most trials rated as low risk of bias. Beta-blockers are highly effective in HFrEF but offer limited benefits in post-MI patients with preserved EF. These findings support phenotype-specific therapy, reinforcing guideline recommendations for HFrEF while questioning routine use in preserved EF post-MI.

## Introduction and background

Heart failure with reduced ejection fraction (HFrEF) remains a major global public health challenge, characterized by impaired ventricular contractility and insufficient cardiac output to meet metabolic demands [1]. Despite advances in diagnostic and therapeutic strategies, HFrEF continues to be associated with high morbidity, recurrent hospitalizations, and significant mortality [2]. The pathophysiology of HFrEF involves complex neurohormonal activation, including sympathetic overdrive and renin-angiotensin-aldosterone system (RAAS) stimulation, which initially compensates for declining cardiac function but ultimately drives maladaptive ventricular remodeling and disease progression [3].

Beta-adrenergic blockers (beta-blockers) have emerged as a cornerstone in the pharmacological management of HFrEF [4]. By antagonizing beta-adrenergic receptors, these agents attenuate the deleterious effects of chronic sympathetic stimulation, leading to improved myocardial energy efficiency, reduced heart rate, and reversal of pathological remodeling [5]. Multiple landmark randomized controlled trials (RCTs) have demonstrated that beta-blockers not only improve left ventricular ejection fraction (LVEF) but also reduce all-cause mortality and hospitalizations in patients with HFrEF. These benefits have been observed across diverse populations, varying etiologies, and multiple beta-blocker subtypes, including bisoprolol, carvedilol, and metoprolol succinate [6].

While current international guidelines strongly recommend beta-blockers as part of the foundational quadruple therapy for HFrEF, clinical practice still exhibits variability in drug selection, dosing strategies, and patient adherence [7]. Furthermore, differences in trial designs, patient demographics, comorbidities, and follow-up durations may influence the interpretation of efficacy and safety outcomes. In light of these considerations, a comprehensive synthesis of high-quality RCT evidence is essential to clarify the magnitude of benefit, highlight potential limitations, and identify gaps for future research.

This systematic review aims to critically evaluate the evidence from RCTs investigating the effects of beta-blockers in patients with HFrEF, focusing on clinical endpoints such as all-cause mortality, heart failure hospitalizations, and changes in LVEF. By integrating and comparing data from diverse RCTs, this review seeks to provide a nuanced understanding of the therapeutic role of beta-blockers in this high-risk population.

## Review

Methodology

This systematic review was conducted in accordance with the Preferred Reporting Items for Systematic Reviews and Meta-Analyses (PRISMA) guidelines [8].

Eligibility Criteria

Only RCTs investigating the efficacy and/or safety of beta-blockers in patients diagnosed with HFrEF were included. Eligible studies were required to report at least one of the following outcomes: all-cause mortality, heart failure-related hospitalizations, or changes in LVEF. Studies were excluded if they were non-randomized, observational, case series, review articles, animal studies, or involved patients with preserved EF or acute decompensated heart failure.

Information Sources and Search Strategy

A comprehensive literature search was conducted using ClinicalTrials.gov, PubMed, and Scopus databases to identify relevant RCTs. No restrictions on publication date or language were applied to ensure an exhaustive retrieval of evidence. The search strategy combined controlled vocabulary (MeSH terms) and free-text terms related to beta-blockers and HFrEF. Boolean operators ("AND" and "OR") were used to enhance sensitivity. The search was last updated on 20 July 2025. The reference lists of included studies and relevant review articles were also screened manually to identify additional eligible trials.

Study Selection

All retrieved records were imported into EndNote version 21 (Clarivate Analytics) for duplicate removal. Two independent reviewers screened the titles and abstracts of the remaining studies to identify potentially eligible trials. Full-text screening was then performed for shortlisted articles to confirm eligibility according to the predefined inclusion criteria. Any discrepancies between reviewers were resolved through discussion or consultation with a third reviewer.

Data Extraction

Data were independently extracted by two reviewers using a standardized data extraction form. Extracted variables included: study characteristics (author, year, country), patient demographics and baseline characteristics, inclusion criteria, beta-blocker type and dosing regimen, comparator intervention, duration of follow-up, and reported outcomes (all-cause mortality, HF hospitalizations, LVEF change, and adverse events). Disagreements were resolved by consensus.

Risk of Bias Assessment

The methodological quality of included RCTs was assessed using the Cochrane RoB 2 tool [9]. This tool evaluates potential bias across five domains: (1) randomization process, (2) deviations from intended interventions, (3) missing outcome data, (4) measurement of the outcome, and (5) selection of the reported results. Each domain was rated as "low risk," "some concerns," or "high risk." Assessments were conducted independently by two reviewers, with disagreements resolved by discussion.

Data Synthesis and Rationale for Narrative Approach

A quantitative meta-analysis was not performed due to substantial heterogeneity in study designs, patient populations, intervention protocols (type and dose of beta-blockers), follow-up durations, and outcome measurement methods. These variations precluded valid statistical pooling and would risk producing misleading summary estimates. Instead, a narrative synthesis was undertaken, structured around key clinical outcomes and methodological features of the included trials, allowing for a more contextually accurate interpretation of the evidence.

Results

Study Selection Process

**Figure 1:**
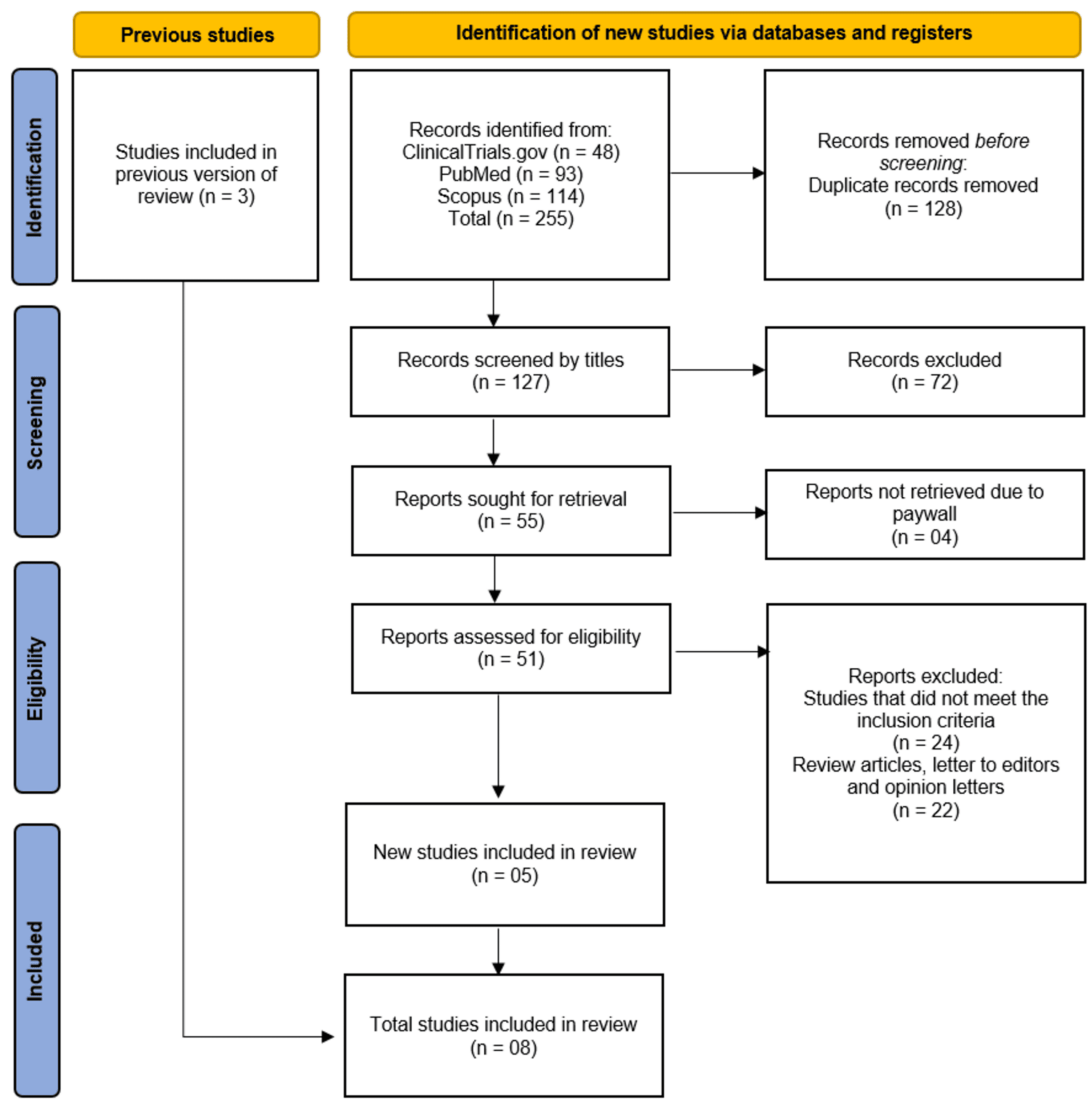
PRISMA flowchart of study selection process PRISMA, Preferred Reporting Items for Systematic Reviews and Meta-Analyses

During the study selection process, three eligible studies were carried forward for inclusion from a previous systematic review [10]. New searches across ClinicalTrials.gov (n=48), PubMed (n=93), and Scopus (n=114) yielded 255 records, from which 128 duplicates were removed. After screening 127 unique records by title/abstract, we excluded 72 irrelevant studies. Full-text assessment was performed for 55 articles, with four unavailable due to paywall restrictions. We excluded 46 additional records (24 failing inclusion criteria; 22 being reviews/letters). In total, eight studies [11-18] qualified for inclusion, comprising the three original studies [11-13] from the prior review and five newly identified studies [14-18] from our updated search (Figure 1).

Characteristics of Included Studies

The systematic review included eight RCTs [11-18] evaluating the efficacy and safety of beta-blockers in patients with HFrEF or post-myocardial infarction (MI) populations. The studies varied in design, sample size, patient demographics, and intervention protocols (Table 1).

**Table 1:** Characteristics of included RCTs EF, ejection fraction; MI, myocardial infarction; RCT, randomized controlled trial; STEMI, ST-segment elevation myocardial infarction; NSTEMI, non-ST-segment elevation myocardial infarction

Author (year)	Country/region	Study design	Sample size (n)	Patient population/inclusion criteria	Intervention (beta-blocker type, dose)	Comparator (placebo/other drug)	Duration of follow-up	Primary outcomes	Secondary outcomes
Watanabe et al. [11] (2018)	Japan	Multicenter, open-label, RCT	801 (Carvedilol group: 399; No beta-blocker group: 402)	STEMI patients with successful primary PCI within 24 hours of onset, LVEF ≥40%, and no heart failure, LV dysfunction, or ventricular arrhythmia	Carvedilol, starting dose 3.4±2.1 mg, up-titrated to 6.3±4.3 mg at 1 year	No beta-blocker	Median 3.9 years (96.4% follow-up)	Composite of all-cause death, MI, hospitalization for heart failure, and hospitalization for acute coronary syndrome	Any coronary revascularization; LVEF at 1 year
Yndigegn et al. [12] (2024)	Sweden (38 centers), Estonia (1 center), New Zealand (6 centers)	Registry-based, randomized, open-label	5020	Age ≥18 years; type I STEMI or NSTEMI ≤7 days prior; coronary angiography during index hospitalization; obstructive CAD (stenosis ≥50% or positive invasive physiologic testing); LVEF ≥50%	Oral metoprolol (target ≥100 mg daily) or bisoprolol (target ≥5 mg daily), initiated inpatient and continued post-discharge	Usual care (beta-blocker tapered off if already on therapy)	Median 3.5 years	Composite of all-cause death or nonfatal AMI: HR 0.96 (95% CI 0.79-1.16), p=0.64	All-cause death: HR 0.94 (p=0.66); AMI: HR 0.96 (p=0.74); HF hospitalization: HR 0.91 (p=0.76); safety outcomes (bradyarrhythmia, hypotension, syncope, asthma/COPD, stroke) - no significant differences
Silvain et al. [13] (2024)	France	Randomized, parallel, open-label	3698 (interruption: 1846; continuation: 1852)	MI ≥6 months before enrollment; current beta-blocker use; median age 64 years; 17% female; 20% with diabetes; median LVEF 60%; median time from MI to randomization 2.9 years; 95% revascularization for index MI. Exclusion: chronic HF, LVEF <40%, cardiac event within last 6 months, other indication for beta-blocker (arrhythmia, migraine, hypertension)	Beta-blocker interruption (type/dose not specified)	Beta-blocker continuation (type/dose not specified)	Median 3 years	Composite of death, MI, stroke, or hospitalization for a cardiovascular reason	Death, MI, stroke, hospitalization for cardiovascular reason, quality of life
CR [14] (1999)	Multicenter	Double-blind RCT (preceded by 2-week single-blind placebo run-in)	3991	Chronic heart failure, NYHA class II-V, LVEF ≤0.40, stabilized on optimum standard therapy	Metoprolol CR/XL: initial 12.5 mg (NYHA III-IV) or 25 mg (NYHA II) once daily, target 200 mg once daily, up-titrated over 8 weeks	Placebo	Mean follow-up 1 year	All-cause mortality	Sudden death, death from worsening heart failure, safety/tolerability
Packer et al. [15] (2001)	334 centers in 21 countries	Randomized, double-blind, placebo-controlled trial	2289 (Carvedilol: 1156; placebo: 1133)	Patients with symptoms of heart failure at rest or on minimal exertion, clinically euvolemic, EF <25%; excluded if requiring intensive care, with marked fluid retention, or on IV vasodilators/inotropic drugs	Carvedilol (dose not specified in abstract)+standard HF therapy	Placebo + standard HF therapy	Mean 10.4 months	All-cause mortality	Combined endpoint of death or hospitalization; withdrawals due to adverse effects; subgroup analyses
Poole-Wilson et al. [16] (2003)	Multicenter, international	Double-blind, randomized, parallel-group trial	1511 (carvedilol group) + 1518 (metoprolol group) = 3029 total	Chronic heart failure (NYHA II-IV), previous cardiovascular admission, LVEF <0.35, on optimal diuretics & ACE inhibitors (unless not tolerated)	Carvedilol, target dose 25 mg twice daily	Metoprolol tartrate, target dose 50 mg twice daily	Mean 58 months (SD 6)	All-cause mortality; composite endpoint of all-cause mortality or all-cause admission	Side effects; drug withdrawals; subgroup analyses of mortality reduction
Investigators [17] (2001)	Multinational, demographically diverse	Randomized, double-blind, placebo-controlled trial	2708 (Bucindolol: 1354; placebo: 1354)	NYHA functional class III (92%) or IV (8%), LVEF ≤35%	Bucindolol (dose not specified)	Placebo	Average 2.0 years	Death from any cause	Death from cardiovascular causes, heart transplantation, or death; subgroup survival benefit in nonblack patients
Flather et al. [18] (2005)	Multinational	Randomized, placebo-controlled trial	2128 (Nebivolol: 1067; placebo: 1061)	Patients ≥70 years with history of HF (hospital admission for HF within previous year or LVEF ≤35%)	Nebivolol, titrated from 1.25 mg once daily to 10 mg once daily (mean maintenance dose: 7.7 mg)	Placebo (mean maintenance dose: 8.5 mg)	Mean 21 months	Composite of all-cause mortality or cardiovascular hospital admission (time to first event)	All-cause mortality; subgroup analyses by age, gender, and EF

Three studies focused on post-MI patients with preserved or mildly reduced EF. Watanabe et al. [11] investigated carvedilol in ST-segment elevation MI (STEMI) patients with EF ≥40%, while Yndigegn et al. [12] and Silvain et al. [13] examined beta-blocker continuation versus interruption in MI patients with EF ≥50% and ≥40%, respectively. The remaining five trials enrolled chronic HFrEF patients with EF ≤35-40%. These included the Metoprolol CR/XL trial [14], the Carvedilol Prospective Randomized Cumulative Survival (COPERNICUS) trial [15], the Carvedilol Or Metoprolol European Trial (COMET) [16], the Beta-Blocker Evaluation of Survival Trial (BEST) [17], and the Study of Effects of Nebivolol Intervention on Outcomes and Rehospitalisation in Seniors (SENIORS) [18]. Sample sizes ranged from 801 [11] to 5020 [12], with follow-up durations spanning one to 5.8 years.

Primary Outcomes

Beta-blockers demonstrated significant mortality benefits in chronic HFrEF but not in post-MI patients with preserved EF. In the COPERNICUS trial, carvedilol reduced all-cause mortality by 35% (95% CI: 19-48%; p=0.0014) compared to placebo [15]. Similarly, metoprolol CR/XL showed a 34% relative risk reduction in mortality (RR 0.66, 95% CI: 0.53-0.81; p=0.00009) [14], and nebivolol reduced the composite of mortality or cardiovascular hospitalization (HR 0.86, 95% CI: 0.74-0.99; p=0.039) [18]. COMET reported superior survival with carvedilol over metoprolol (HR 0.83, 95% CI: 0.74-0.93; p=0.0017) [16].

In contrast, beta-blockers did not significantly improve primary endpoints in post-MI patients with preserved EF. Watanabe et al. [11] found no difference in composite outcomes (6.8% vs. 7.9%; p=0.20), while Yndigegn et al. [12] reported a neutral effect (HR 0.96, 95% CI: 0.79-1.16; p=0.64). Silvain et al. [13] confirmed noninferiority of beta-blocker interruption (23.8% vs. 21.1%; p=0.44 for noninferiority).

Secondary Outcomes

Heart failure hospitalizations were reduced in HFrEF trials. Packer et al. [15] observed a 24% reduction in combined death or hospitalization, and COMET reported a trend toward fewer admissions (HR 0.94, 95% CI: 0.86-1.02) [16]. However, no significant differences were noted in post-MI trials [12,13]. Changes in LVEF were modest, with Watanabe et al. [11] reporting a nonsignificant 1.3% improvement at one year (p=0.06).

Safety and Tolerability

Beta-blockers were generally well tolerated. Adverse events, such as bradyarrhythmia and hypotension, were comparable between groups in Yndigegn et al. [12] (HR 1.08, 95% CI: 0.79-1.46; p=0.64). Packer et al. [15] noted fewer withdrawals due to adverse effects with carvedilol (p=0.02). No excess risk of asthma/COPD or stroke hospitalizations was observed [15].

Summary of Evidence

The results are summarized in Table 2. Beta-blockers consistently improved survival and reduced hospitalizations in HFrEF but showed neutral effects in post-MI patients with preserved EF. Tolerability profiles were favorable across studies.

**Table 2:** Summary of results and clinical outcomes from included RCTs RCT, randomized controlled trial

Author (year)	Primary endpoint	Effect on primary endpoint (effect size, 95% CI)	p-value	All-cause mortality (% or HR)	HF hospitalizations (% or HR)	Change in LVEF (%)	Adverse events (n or %)
Watanabe et al. [11] (2018)	Composite of all-cause death, MI, hospitalization for HF, and hospitalization for ACS	6.8% (carvedilol) vs. 7.9% (no beta-blocker), effect size/CI not reported	0.2	NR	NR	60.9±8.4% vs. 59.6±8.8% at 1 year, Δ=+1.3%, p=0.06	NR
Yndigegn et al. [12] (2024)	Composite of all-cause death or nonfatal AMI	HR 0.96 (95% CI 0.79-1.16)	0.64	3.9% vs. 4.1%, HR 0.94 (95% CI 0.71-1.24), p=0.66	0.8% vs. 0.9%, HR 0.91 (95% CI 0.50-1.66), p=0.76	NR	Hospitalization for bradyarrhythmia, hypotension, or syncope: 3.4% vs. 3.2%, HR 1.08 (95% CI 0.79-1.46), p=0.64; asthma/COPD hospitalization: 0.6% vs. 0.6%, HR 0.94 (95% CI 0.46-1.89), p=0.86; stroke hospitalization: 1.4% vs. 1.8%, HR 0.78 (95% CI 0.51-1.21), p=0.35
Silvain et al. [13] (2024)	Composite of death, MI, stroke, or hospitalization for a cardiovascular reason	23.8% (interruption) vs. 21.1% (continuation); p for noninferiority=0.44	0.44 (noninferiority)	4.1% (interruption) vs. 4.0% (continuation)	Only CV hospitalization: 18.9% vs. 16.6%	Median LVEF at baseline: 60%	NR
CR [14] (1999)	All-cause mortality	RR 0.66 (95% CI 0.53-0.81)	p=0.00009 (adjusted p=0.0062)	Metoprolol: 7.2% vs. placebo: 11.0%	NR	NR	Well tolerated
Packer et al. [15] (2001)	All-cause mortality	35% relative risk reduction (95% CI 19-48%)	0.0014	Placebo: 190 deaths/1133; carvedilol: 130 deaths/1156	Combined death or hospitalization reduced by 24% (numerical HF hospitalization rate not specified)	NR	Fewer withdrawals due to adverse effects in carvedilol group (P=0.02)
Poole-Wilson et al. [16] (2003)	All-cause mortality	HR 0.83 (95% CI 0.74-0.93)	0.0017	Carvedilol: 34% (512/1511) vs. Metoprolol: 40% (600/1518)	Composite endpoint (mortality or all-cause admission): HR 0.94 (95% CI 0.86-1.02)	NR	Incidence of side effects and drug withdrawals similar between groups
Investigators [17] (2001)	Death from any cause	No significant difference; mortality 33% (placebo) vs. 30% (bucindolol); secondary: CV death HR 0.86 (95% CI 0.74-0.99)	0.13 (adjusted), 0.16 (unadjusted)	33% (placebo) vs. 30% (bucindolol)	NR	NR	NR
Flather et al. [18] (2005)	Composite of all-cause mortality or cardiovascular hospital admission (time to first event)	HR 0.86 (95% CI 0.74-0.99)	0.039	15.8% (nebivolol) vs. 18.1% (placebo), HR 0.88 (95% CI 0.71-1.08)	NR	NR	NR

Risk of Bias Assessment Results

The Cochrane RoB 2 tool was applied to assess the methodological quality of the included RCTs. Most studies demonstrated low risk of bias across all domains, including randomization process, deviations from intended interventions, missing outcome data, measurement of the outcome, and selection of reported results [12-18]. However, Watanabe et al. [11] raised some concerns, primarily due to its open-label design, which introduced potential bias in the randomization process and deviations from intended interventions, though other domains remained low risk (Table 3). Collectively, the evidence from these trials is highly reliable, with only minor limitations in one study.

**Table 3:** Risk of bias assessment on Cochrane RoB 2 tool

Study (year)	Randomization process	Deviations from intended interventions	Missing outcome data	Measurement of the outcome	Selection of reported results	Overall risk of bias
Watanabe et al. [11] (2018)	Some concerns	Some concerns	Low risk	Low risk	Low risk	Some concerns
Yndigegn et al. [12] (2024)	Low risk	Low risk	Low risk	Low risk	Low risk	Low risk
Silvain et al. [13] (2024)	Low risk	Low risk	Low risk	Low risk	Low risk	Low risk
CR [14] (1999)	Low risk	Low risk	Low risk	Low risk	Low risk	Low risk
Packer et al. [15] (2001)	Low risk	Low risk	Low risk	Low risk	Low risk	Low risk
Poole-Wilson et al. [16] (2003)	Low risk	Low risk	Low risk	Low risk	Low risk	Low risk
Investigators [17] (2001)	Low risk	Low risk	Low risk	Low risk	Low risk	Low risk
Flather et al. [18] (2005)	Low risk	Low risk	Low risk	Low risk	Low risk	Low risk

Discussion

The findings of this systematic review, encompassing eight RCTs, provide a comprehensive evaluation of beta-blockers in HFrEF and post-MI populations. The results underscore a clear dichotomy: beta-blockers demonstrate significant mortality and morbidity benefits in chronic HFrEF but exhibit neutral effects in post-MI patients with preserved or mildly reduced EF. This distinction is critical for clinical practice, as it reinforces the established role of beta-blockers in HFrEF while challenging their blanket use in post-MI patients without systolic dysfunction.

The mortality reduction observed in HFrEF trials aligns robustly with existing literature. The COPERNICUS trial [15] reported a 35% relative risk reduction in all-cause mortality with carvedilol, a finding consistent with earlier meta-analyses demonstrating class-specific benefits of beta-blockers in HFrEF [19]. Similarly, the Metoprolol CR/XL trial [14] and COMET [16] reinforced these results, with the latter suggesting carvedilol’s superiority over metoprolol tartrate, a finding that has sparked debate about the role of beta-1 selectivity and ancillary properties in clinical outcomes [16]. The SENIORS trial [18] further extended these benefits to elderly patients, a subgroup often underrepresented in clinical trials, by showing nebivolol’s efficacy in reducing composite endpoints. These results collectively validate current guideline recommendations favoring beta-blockers as cornerstone therapy in HFrEF [20].

In contrast, the neutral effects of beta-blockers in post-MI patients with preserved EF, as seen in Watanabe et al. [11], Yndigegn et al. [12], and Silvain et al. [13], challenge long-standing assumptions. The absence of mortality or hospitalization benefits in these studies suggests that the mechanisms driving beta-blocker efficacy in HFrEF, such as attenuation of sympathetic overdrive and ventricular remodeling, may not translate to patients with intact systolic function. This is particularly evident in Yndigegn et al. [12], where metoprolol or bisoprolol showed no advantage over usual care, and in Silvain et al. [13], where beta-blocker interruption was noninferior to continuation. These findings resonate with emerging literature questioning the routine use of beta-blockers in MI patients without HFrEF [21], and they highlight the need for precision medicine approaches tailored to the underlying pathophysiology.

The secondary outcomes further refine our understanding. The reduction in heart failure hospitalizations in HFrEF trials, such as the 24% decrease observed by Packer et al. [15], underscores beta-blockers’ role in mitigating disease progression. However, the lack of similar benefits in post-MI trials [12,13] suggests that hospitalization drivers in these populations may differ, potentially involving non-cardiac comorbidities or non-hemodynamic mechanisms. The modest changes in LVEF, exemplified by Watanabe et al. [11], also imply that beta-blockers’ benefits in HFrEF may stem more from neurohormonal modulation than from sheer improvement in systolic function, a hypothesis supported by mechanistic studies [22].

Safety and tolerability profiles were uniformly favorable across studies, with no significant excess in adverse events. The low withdrawal rates in trials like COPERNICUS [15] and COMET [16] reflect careful titration protocols and patient selection, while the absence of increased asthma/COPD or stroke hospitalizations in Yndigegn et al. [12] alleviates historical concerns about beta-blocker safety in comorbid populations. These findings align with real-world data confirming the tolerability of guideline-directed beta-blocker therapy [23], though they also underscore the importance of individualized dosing and monitoring.

The risk of bias assessment bolsters confidence in these conclusions. The predominance of low-risk studies, particularly double-blind trials like COMET [16] and SENIORS [18], ensures robust internal validity. The lone study with some concerns, Watanabe et al. [11], employed an open-label design but maintained rigorous outcome adjudication, minimizing the impact of bias. This methodological rigor aligns with Cochrane standards and strengthens the review’s conclusions [24].

When contextualized with existing literature, these findings invite reflection on evolving therapeutic paradigms. The mortality benefits in HFrEF echo those seen in landmark trials like MERIT-HF [25], while the neutral post-MI data resonate with recent critiques of beta-blocker overuse [26]. Notably, our results contrast with older observational studies advocating beta-blockers for all post-MI patients [27], highlighting the pitfalls of extrapolating data from HFrEF to preserved EF populations. This discrepancy underscores the importance of contemporary RCTs in refining clinical practice.

Limitations

Despite its strengths, this review has limitations. First, the heterogeneity in beta-blocker types and dosing regimens complicates cross-trial comparisons. Second, the exclusion of non-English studies and unpublished data may introduce selection bias. Third, the open-label design of some trials raises potential performance bias, though its impact on hard endpoints like mortality is likely minimal. Finally, the focus on RCTs, while methodologically sound, may overlook real-world effectiveness data.

## Conclusions

This study reaffirms beta-blockers’ life-saving role in HFrEF while questioning their utility in post-MI patients with preserved EF. The consistency of mortality benefits in HFrEF across trials, coupled with neutral outcomes in preserved EF populations, calls for a nuanced, phenotype-specific approach to beta-blocker therapy. Future research should explore the mechanisms underlying these differential effects and investigate personalized treatment algorithms. For now, clinicians should adhere to evidence-based guidelines for HFrEF while reconsidering reflexive beta-blocker use in post-MI patients without systolic dysfunction.
